# cGMP Signaling, Phosphodiesterases and Major Depressive Disorder

**DOI:** 10.2174/157015911798376271

**Published:** 2011-12

**Authors:** Gillian W Reierson, Shuyu Guo, Claudio Mastronardi, Julio Licinio, Ma-Li Wong

**Affiliations:** 1 University of Miami Miller School of Medicine, Miami, FL, USA; 2 Department of Translational Medicine, John Curtin School of Medical Research, Australian National University, Acton, ACT, Australia

**Keywords:** Major Depression, cyclic guanosine monophosphate, neurogenesis, neuroplasticity, phophodiesterases, cyclases, antidepressants, pharmacology.

## Abstract

Deficits in neuroplasticity are hypothesized to underlie the pathophysiology of major depressive disorder (MDD): the effectiveness of antidepressants is thought to be related to the normalization of disrupted synaptic transmission and neurogenesis. The cyclic adenosine monophosphate (cAMP) signaling cascade has received considerable attention for its role in neuroplasticity and MDD. However components of a closely related pathway, the cyclic guanosine monophosphate (cGMP) have been studied with much lower intensity, even though this signaling transduction cascade is also expressed in the brain and the activity of this pathway has been implicated in learning and memory processes. Cyclic GMP acts as a second messenger; it amplifies signals received at postsynaptic receptors and activates downstream effector molecules resulting in gene expression changes and neuronal responses. Phosphodiesterase (PDE) enzymes degrade cGMP into 5’GMP and therefore they are involved in the regulation of intracellular levels of cGMP. Here we review a growing body of evidence suggesting that the cGMP signaling cascade warrants further investigation for its involvement in MDD and antidepressant action.

## 1 INTRODUCTION

Major depressive disorder (MDD) is a chronic and highly disabling condition. It is the most common mood disorder: up to 17% of the population suffers from this condition at some point during their lifetimes. While antidepressants have been shown to be effective in treating the symptoms of MDD in 60-70% of patients, 30-40% of patients do not respond to pharmacotherapy [1, 2]. Furthermore, antidepressants must be taken chronically for several weeks before treatment response is achieved and it is still unclear what are the mechanisms by which these drugs relieve the clinical symptoms of MDD. Evidence from clinical and preclinical research accumulated in recent years support the concept that aberrant neuroplasticity, including functional synaptic plasticity and structural plasticity, in the hippocampus and prefrontal cortex, might underlie MDD [3, 4]. Intracellular cyclic adenosine monophosphate (cAMP) signaling activated by brain-derived neurotrophic factor (BDNF) has been shown to be a pivotal player in neuroplasticity and MDD; moreover, studies have demonstrated that treatment with antidepressants can alter the expression of components of this signaling pathway in rodents [5-7]. Unfortunately, earlier findings have been inconsistently replicated making it premature to conceptualize translational strategies for new antidepressant pharmacotherapy targeting the cAMP signaling. The cyclic guanosine monophosphate (cGMP) signaling pathway is an intracellular nucleotide cascade that is also involved in neuroplasticity. It is closely related to cAMP signaling, but it has received little attention in the biology of MDD and antidepressant action. The cGMP signaling cascade consists of a number of different elements that can regulate cGMP levels and influence downstream cellular responses. Fig. (**1**) depicts the presynaptic and postsynaptic elements of the cGMP signaling pathway in the central nervous system; these elements are expressed in the hippocampus, a brain region that has been implicated in MDD [8, 9]. Pharmacotherapy targeting different elements of the cGMP pathway is currently available; this class of drugs includes phosphodiesterase inhibitors, which increase cGMP signaling. In this review, we summarize the evidence that supports the role of cGMP signaling in neuroplasticity and in the pathophysiology of MDD and antidepressant treatment response, with a specific focus on the role of phosphodiesterases (PDEs).

## 2 cGMP SIGNALING COMPONENTS AND THEIR DISTRIBUTION IN THE BRAIN

### 2.1 cGMP Synthesis: pGC and sGC

The synthetic enzyme guanylate/guanylyl cyclase (GC) converts guanosine triphosphate (GTP) to 3’-5’-cyclic guanosine monophosphate to produce the second messenger molecule known as cGMP. The production of cGMP by GC can occur in two different ways: 1) following activation of membrane bound guanylate cyclase (particulate GC or pGC) [10, 11] and 2) following activation of soluble guanylate cyclase (sGC, also called NO receptors) in the cytoplasm by the diffusible second messenger molecule, nitric oxide (NO) [12, 13].

Particulate GC is composed of two subfamilies: the peptide hormone receptor subfamily, which binds natriuretic peptides, and the Ca^2+^-modulated ROS-GC (rod outer segment-derived guanylate cyclase) subfamily, which is linked with the senses of vision, olfaction and taste, and light-regulated pinealocytes [1, 14-16]. The isoforms of pGC expressed in the brain include guanylate cyclase A/natriuretic peptide receptor 1 (GC-A/Npr1), guanylate cyclase B/natriuretic peptide receptor 2 (GC-B/Npr2), guanylate cyclase 2D (GC-D/GUCY2D); while two eye-specific GC have been identified in mammals (designed GC-E and GC-F or RetGC-1 and RetGC-2 in humans) [17-19]. GC-A/Npr1 is activated by atrial natriuretic peptide (ANP) and brain natriuretic peptide (BNP) and the mRNA is expressed in the subfornical organ, medial habenula, cerebellum and olfactory bulb [20, 21]. GC-B/Npr2 is activated by natriuretic peptide type C (CNP) and the mRNA is expressed in the pineal gland, cerebellum, cortex, hippocampus, hypothalamus, olfactory bulb, neural lobe of the pituitary gland, and thalamus [21, 22]. GC-D is expressed in the olfactory bulb in rodents, but it is a pseudogene in humans [23].

Soluble GC exists as a heterodimer composed of different combinations of α subunits (α1 or α2) and β subunits (β1 or β2); all sGC subunits mRNAs are expressed in the brain with the exception of the β2 subunit mRNAs [24]. Both α1 and α2 sGC subunits mRNAs are expressed in the cerebral cortex and olfactory bulb; α1 is also expressed in striatum and pallidum and α2 is also expressed in hippocampus and medulla [21]. The β1 sGC subunit mRNA is expressed throughout the brain, with predominant expression in the cerebral cortex, hippocampus, lateral septal complex, olfactory bulb, and striatum [21, 24]. In the hippocampus, the sGC heterodimer is composed of α2 and β1 subunits [25]. 

### 2.2 cGMP Degradation: PDEs

Several different enzymes of the phosphodiesterase (PDE) family are capable of cleaving cGMP into 5’-GMP. PDEs are classified as cAMP specific (PDE4, PDE7, and PDE8), cGMP specific (PDE5, PDE6, and PDE9), or dual substrate (PDE1, PDE2, PDE3, PDE10, and PDE11) [26, 27]. Dual substrate PDEs are able to hydrolyze both cAMP and cGMP. The specificity of a dual substrate PDE for each cyclic nucleotide substrate is determined by the orientation of an invariant glutamine in the binding pocket of the catalytic domain of the PDE, a concept that is termed “glutamine switch” [28-30]. The orientation of the glutamine is thought to be determined by the presence of neighboring residues that constrain the rotation of the glutamine to only allow the purine ring of one or the other cyclic nucleotides to bind *via* H bond formation; free rotation of the glutamine allows both substrates to bind [30, 31]. The binding affinities (K_m_ values), the catalytic hydrolyzing activities (V_max_, K_cat_) and the presence of specific domains in the N-terminal region of these genes reveal much information about how these PDEs might be uniquely suited to regulate cyclic nucleotide cross-talk. The presence of N-terminal domains is especially influential, as activity in these domains can cause conformational changes in the catalytic domain of the PDE, changing the K_m_ and V_max_ of the enzyme toward cyclic nucleotide substrates [30, 32].

In the following two paragraphs, we will summarize the CNS expression of cGMP specific PDEs (PDE5, PDE6, PDE9) and dual substrate PDEs (PDE1, PDE2, PDE3, PDE10, and PDE11). 

All cGMP specific PDEs are expressed in the brain. In the rodent brain, PDE5A mRNA expression has been reported in the purkinje cells of the cerebellum; strong staining has also been observed in scattered cells in the hippocampus, including pyramidal cells of CA1, CA2 and CA3, as well as in the dentate gyrus [33]. PDE6 was initially thought to be limited to the retina; however, PDE6B mRNA expression has also been reported in mouse hippocampus [34]. CNS expression of the PDE9A mRNA in the rodent brain has been reported in the purkinje cells and granule cells of the cerebellum, olfactory bulb and tubercle, caudate putamen, and CA1 and dentate gyrus areas of the hippocampus [35-37]. In the human brain, PDE9 mRNA expression has been reported in the insular and visual cortices as well as in the CA1, CA2 and CA3 subfields, and dentate gyrus of the hippocampal formation [38]. 

All dual substrate cGMP are also expressed in the brain. In rodents, *in* *situ* hybridization and immunohistochemistry studies demonstrated that the PDE1A isoform is expressed in the following brain areas: cerebral cortex, pyramidal cells of the hippocampus, and striatum [39, 40]. PDE1B is also expressed in several brain areas including the caudate-putamen, nucleus accumbens, dentate gyrus of hippocampus, olfactory tubercle, medial thalamic nuclei, and brainstem [39, 40]. PDE1C mRNA is expressed in the granule cells of the cerebellum, caudate-putamen, olfactory tubercle, and brainstem of the rodent brain [41]. In the human brain, hippocampal PDE1B expression has been reported in the granule cells of the dentate gyrus and in pyramidal cells [42]. PDE2 mRNA is expressed in the rodent medial habenula, olfactory bulb and tubercle, cortex, amygdala, striatum, and hippocampus [33]. Within the rodent hippocampus, PDE2 protein is expressed in the pyramidal cells of CA1 to CA3 subfields and in the granule cells of the dentate gyrus [37]. In the human brain, PDE2 mRNA expression has been found in the insular and visual cortices as well as in the hippocampal formation [38]. In a systematic immunohistochemistry study, PDE2A protein was expressed in the limbic system, including hippocampus, basal ganglia, amygdala, isocortex, habenula, and interpeduncular nucleus [43]. The mRNAs of both PDE3A and PDE3B isoforms are expressed in the rodent hippocampus, with PDE3A also displaying expression in the striatum and PDE3B displaying expression in the cerebellum [44]. According to immunohistochemistry studies, PDE10A is expressed in the pyramidal cells and dentate gyrus of the hippocampus, cortex, granule cells of the cerebellum, and is especially enriched in the striatum [45-47]. The mRNA and protein of PDE11A are expressed in the trigeminal ganglion, neocortex, spinal trigeminal nucleus, and purkinje cells of the cerebellum of rats [48]. In the human brain, PDE11A4 protein is expressed in the pituitary [49].

### 2.3 cGMP Downstream Effectors: PKG/cGK

The downstream effectors of cGMP include protein kinases, cyclic nucleotide gated channels (CNG), and cAMP specific PDEs. The major downstream effector of cGMP is the protein kinase G (PKG), which is alternatively referred to as cyclic GMP dependent protein kinase (cGK). PKGs belong to a family of serine/threonine kinases because they phosphorylate target protein substrates at specific structural motifs. There are two different PKG genes, *PRKG1 *and *PRKG2* that encode cytosolic PKGI/cGKI and membrane-bound PKGII/cGKII, respectively. PKGI can be alternatively spliced at the N-terminal into two isoforms, PKGIα and PKGIβ, varying in tissue distribution, subcellular localization, and substrate interaction [50].

PKG is comprised of three different functional domains: N-terminal domain containing regulatory sites, a regulatory domain containing cGMP binding sites, and a catalytic domain in the C-terminal region. The regulatory sites of the N-terminal domain of PKG include domains that influence dimerization, autoinhibitory, autophosphorylation, affinity and cooperative behavior, and intracellular localization. Upon binding of two molecules of cGMP to the two binding sites present in the regulatory domain of PKG, the autoinhibitory site that normally inhibits the catalytic domain of PKG is removed, thus activating the protein. The catalytic domain of PKG is located at the C-terminal end and contains binding sites for Mg^2+^, ATP, and the target protein. Once activated, PKG phosphorylates target proteins at RKRKXST structural motifs [51].

Although PKGI was previously reported to display limited expression restricted to the Purkinje cells of the cerebellum, neurons in the nigrostriatal pathway, and dorsomedial nucleus of the hypothalamus, further studies now demonstrate that PKGI is expressed in additional brain areas such as the hippocampus, olfactory bulb and amygdala [21, 52, 53]. PKGI expression has been found in the following mouse brain structures: cerebellum, hippocampus, dorsomedial hypothalamus, medulla, subcommisural organ, cerebral cortex, amygdala, habenula, hypothalamus, olfactory bulb, pituitary, and retina, with isoform specific PKGIα expression in the cerebellum and medulla and PKGIβ expression in the cortex, hippocampus, hypothalamus, and olfactory bulb [9]. PKGII is widely expressed throughout the rat brain, in structures including the cerebral cortex, cerebellum, and brainstem with greater expression in the neuropil as compared to cell bodies [10]. PKGII expression has been also reported in thalamus, outer layers of cerebral cortex, septum, amygdala, and olfactory bulb [21, 53].

## 3 REGULATION OF cGMP SIGNALING

### 3.1 Compartmentalization and Crosstalk: Two Important Concepts 

A variety of extracellular cues are able to initiate common intracellular signaling cascades, such as the cGMP signal transduction pathway, to produce distinct biological effects. It is unclear how exactly different ligands are capable of producing distinct messages through intracellular signaling cascades resulting in unique cellular responses despite acting through cGMP, which is their common second messenger intermediate. The concept of “compartmentalization” of cyclic nucleotide signaling in time and space has been proposed to account for this discrepancy.

Through the use of fluorescence energy resonance transfer (FRET) techniques in which specially engineered fluorescent probes are used to detect cyclic nucleotide levels in cells, much information has been gained regarding the propagation of the cyclic nucleotide signal in time and space. For example, observations of cAMP signaling in live cells using FRET have demonstrated that the accumulation of this small second messenger occurs in local “cAMP pools” [54]. This observation has led to the idea of “compartmentalization” or the presence of confined compartments/microdomains of cAMP signaling resulting from physical barriers in the cell that restrict the diffusion of cAMP, forming gradients [55]. Macromolecular complexes comprising this physical barrier are composed of the A-Kinase anchoring proteins (AKAPs), which create a scaffold or platform for interactions between regulatory and effector proteins relevant to cAMP signaling also present in the complex [34].

One of the target effectors of cGMP is PKG. PKG is activated when two molecules of cGMP bind to the regulatory subunits of this protein, releasing the autoinhibitory domain of the regulatory subunit from the catalytic subunit. Activated PKG then can go on to phosphorylate any number of different downstream targets to result in distinct cellular responses. Interestingly, PKGs participate in the negative feedback of cGMP signaling by phosphorylating and activating PDEs, the enzymes that degrade cyclic nucleotides, to decrease the levels of cGMP [56]. By regulating the levels of cyclic nucleotides, PDEs are thought to play a critical regulatory role in fine-tuning the cGMP message in both time and space. As PDEs are expressed in specific subcellular locations, it has been proposed that PDEs are strategically placed in cells and act as “sinks” to increase the turnover of cyclic nucleotides and thereby dampen the cyclic nucleotide message [54]. PDEs recruited to macromolecular complexes through their activation by PKG phosphorylation could potentially decrease the amount of cGMP in a distinct subcellular microdomain. Pharmacological PDE inhibitors are hypothesized to result in abnormal cyclic nucleotide signaling by disrupting local cyclic nucleotide pools so that they “spill over” from their confined compartments to inappropriately activate target effectors previously not accessible [55].

The presence of cGMP pools located in specific cellular regions suggests that different biological effects could be produced through the action of different combinations of PKGs and PDEs present in macromolecular complexes at these specialized cyclic nucleotide microdomains [54]. While the details of “compartmentalization” of different cGMP messages need to be clarified, the concept provides an intriguing explanation to the question of how different extracellular cues working through the same intracellular messenger can produce different biological outcomes. Therefore, it is likely that the cGMP message is subject to compartmentalization, with macromolecular complexes composed of AKAPs, PKG, and PDEs confining cGMP into specialized microdomains or localized pools of cGMP [55]. Further studies are needed to determine which AKAPs coordinate cGMP hydrolyzing PDEs and PKG to regulate the amount of cGMP. 

The concept of “crosstalk” in intracellular signaling transduction cascades refers to the ability of components from one signaling pathway to interact with components of another signaling pathway. There is already ample evidence of crosstalk between cAMP and cGMP signaling pathways, with PDEs specifically hypothesized to act as points of intersection between these two pathways [57, 58]. Given the evidence that PDEs are targeted to distinct subcellular locations where they potentially regulate the levels of cyclic nucleotides by acting as “sinks” within “compartmentalized” anchored signaling complexes to define microdomains of cyclic nucleotides within cells, it is plausible that PDEs could also operate as points of crosstalk control in the cell between cAMP and cGMP signaling networks. Two dual substrate PDEs, namely PDE2 and PDE3, have emerged as likely candidates to coordinate the interaction between intracellular cyclic nucleotide signaling pathways [57].

### 3.2 Synthesis Regulation: NO/sGC/cGMP 

Nitric oxide (NO) is a free radical and a small molecule gas that is capable of diffusing across membranes where it can act as a second messenger in signaling cascades. Specifically, NO acts as the intracellular ligand for the soluble guanylate cyclase (sGC or NO receptors), which are cGMP synthetic enzyme present in the cytosol. Upon binding of NO to sGC, cGMP is synthesized from GTP. Therefore, NO is an important regulator of cGMP levels because of its activating effects on sGC. In the nervous system, NO/cGMP signaling mediates long-term potentiation (LTP) and NO was considered as a retrograde messenger [59]. Specifically, activation of NMDA (N-methyl-d-aspartate) receptors have been found to increase postsynaptic synthesis of NO by Ca^2+^/calmodulin dependent neuronal NOS (NO synthase, NOS-1/nNOS). The diffusible NO can then activate sGC that result in the presynaptic production of cGMP and neurotransmitter release. Soluble GCs occurs as two isoforms: NO-GC1 and NO-GC2. Interestingly, it has been reported that both NO-GCs are required for LTP and that NMDA-induced NO increases cGMP only in the presence of NO-GC1, which have implied different synaptic localizations for NO-GC1 and NO-GC2. This evidence indicates that the compartmental regulation of cGMP signaling is involved in synaptic plasticity and that sGC may play an important role in the precise spatial control of cGMP synthesis. 

### 3.3 Degradation Regulation: PDE2, PDE3 and PDE5.

PDE2, 3 and 5 are relevant regulatory PDE’s because they may be activated or inhibited by cGMP and they effect changes in the levels of cGMP or cAMP.

#### PDE2

The binding affinities of PDE2 for cAMP (K_m_=30 µM) and cGMP (K_m_=10 µM) reveal that PDE2 can bind both cyclic nucleotides equally and is capable of hydrolyzing both cAMP and cGMP [57]. However, PDE2 is referred to as the “cGMP-stimulated cAMP PDE” because cGMP binding to GAF (cGMP-biding PDE, *Anabaena* adenylyl cyclases, *Eschericihia coli* FhlAs) domains present in the N-terminal region allosterically stimulates the cAMP hydrolyzing activity of PDE2, resulting in decreased cAMP levels. Cyclic GMP binding to the GAF domain of PDE2 results in a conformational change to the catalytic domain of PDE2, changing the kinetics (decreasing K_m_ and increasing V_max_ values) of PDE2 towards the cAMP substrate, and increasing the hydrolyzing activity of PDE2 specifically towards the breakdown of cAMP into 5’AMP. Interestingly, PDE2A has three splice variants, among which, PDE2A3 is entirely membrane associated in the mouse brain. Moreover, in cultures of hippocampal neurons, PDE2A co-localized with the synaptic marker synaptophysin, in a punctate pattern. Therefore, PDE2A represents a key point of compartmentalized cross-talk between cAMP and cGMP signaling.

#### PDE3

Cyclic AMP and cGMP act as mutually competitive substrates for PDE3, and the binding affinities of PDE3 for cAMP (K_m_=0.08 µM) and cGMP (K_m_=0.02 µM) demonstrate that PDE3 is indeed a dual substrate PDE capable of binding each cyclic nucleotide substrate with similar affinity [57]. However, as PDE3 demonstrates a higher rate of catalysis for cAMP (10- fold higher V_max_) versus cGMP, it is referred to as “cAMP preferring.” PDE3 has also been called the “cGMP-inhibited cAMP PDE” because of *in vitro* experimental evidence that cGMP can inhibit the cAMP hydrolyzing activity of PDE3 to increase the levels of cAMP [60]. Evidence of a putative PKG phosphorylation site in the N-terminal domain of PDE3 indicates that PDE3 activity can be indirectly influenced by cGMP *via* downstream kinase effects of PKG to phosphorylate PDE3 [57]. PDE3 therefore represents a point of crosstalk control, another example of a cAMP hydrolyzing PDE that might be a target of regulation by cGMP-mediated signaling. 

#### PDE5

PDE5 is referred to as the “cGMP-stimulated cGMP PDE,” as the N-terminal region of the PDE5 gene contains a GAF domain where cGMP can bind, as well as a site for phosphorylation by PKG. Cyclic GMP allosteric binding to the GAF domain of PDE5 stimulates the enzyme, decreasing the K_m_ to increase the affinity of the binding site for cGMP as well increasing the catalytic activity of PDE5 to hydrolyse cGMP. By binding to the GAF domain of PDE5, cGMP is thought to increase the accessibility of the Ser102 residue of PDE5 to phosphorylation by PKG, thereby activating the enzyme. There is evidence that PKA might also phosphorylate PDE5, an intriguing example of possible crosstalk regulation between cAMP and cGMP signaling pathways [61].

## 4 The role of CGMP signaling in brain plasticity and MDD

### 4.1 The Neuroplasticity Hypothesis of MDD

Although the currently accepted mechanism of action of available antidepressants suggests the involvement of the monoamine system in MDD, increasing evidence has supported that neuroplasticity is disturbed in this disorder. Neuroplasticity includes functional synaptic plasticity (regulation of synaptic transmission) and structural plasticity (such as adult neurogenesis and permanent morphological changes of synaptic structure). 

Functional synaptic plasticity is mediated by glutamate signaling. Glutamate binds to postsynaptic NMDA receptor, increasing synaptic calcium flux, which induces the activation of kinases that is critical for the early phase of LTP, such as calcium-calmodulin-dependent kinase II (CaMKII). CaMKII can trigger insertion of new AMPA [2-amino-3-(5-methyl-3-oxo-1,2-oxal-4-yl) propanoic acid] receptors in the postsynaptic membrane by phosphorylating the GluR1 (glutamate receptor type 1) subunit of the AMPA receptor, which results in activation of “silent synapsis” and long-term potentiation (LTP) [62]. LTP strengthens synaptic transmission, representing occurrence of memory, while long-term depression (LTD) decreases synaptic function, representing weakening of memory. In experimental animals, severe stress can impair LTP and enhance LTD in the hippocampus [63-65]. Both electroconvulsive shock and chronic antidepressant treatments reverse impaired LTP in animal models of depression [66, 67].

In the late phase of LTP, the accumulation of cAMP and calcium activate signal transduction cascades. PKA, MAPK and CaMKII translocate to the cell nucleus and phosphorylate the transcription factor CREB (cAMP response element-binding), which in turn induces gene transcription and protein synthesis of brain derived neurotrophic factor (BDNF) and vascular endothelial growth factor (VEGF), leading to changes in the synaptic structure [24, 25, 33]. Adult neurogenesis occurs in the dentate gyrus region of the hippocampus and it is supposedly regulated by LTP and activation of CREB. In animal models, chronic stress induces morphologic changes in the hippocampus and prefrontal cortex: the number of dendritic spines and synapses decrease, and neurogenesis in the dentate gyrus is impaired [68, 69]. In clinical research, structural imaging also demonstrated hippocampal atrophy in MDD patients [70, 71]. Electroconvulsive shock and chronic antidepressant treatments can increase neurogenesis, improve the rate of proliferation and the survival of neurogenesis through the cAMP-CREB cascade [72-74].

### 4.2 cGMP and Functional Synaptic Plasticity

Evidence indicates that cGMP signaling plays a pivotal role in LTP. Inhibitors of PKG/sGC can block the induction of LTP. During tetanus induced LTP, PKG/sGC is activated leading to transient increases in cGMP levels and activation of PKG. Cyclic GMP signaling is transduced by down-stream effectors such as CNG, PKGI/cGKI, PKGII/cGKII, and cGMP-activated phosphodiesterases, which underlie the crosstalk between cAMP and cGMP signaling. It has been reported that the presynaptic NO/cGMP signaling induced during LTP facilitates glutamate release *via* hyperpolarization of activated cyclic nucleotide-gated channels, which in turn enhances synaptic transmission [75]. The downstream events of cGMP/PGK signaling are under investigation, but increasing evidence has indicated their critical role in LTP. PKGI/cGKI is colocalized with synaptophysin and concentrated in a punctate pattern, which is activated and phosphorylates presynaptically and postsynaptically VASP (vasodilator-stimulated phosphoprotein) during potentiation, increasing aggregation of synaptic proteins [76]. PKGII/cGKII interact with GluR1 (Glutamate receptor1), a subunit of AMPA (2-amino-3-(5-methyl-3-oxo-1,2- oxazol-4-yl)propanoic acid) receptor, regulating AMPA receptor trafficking, which facilitate the insertion of AMPA receptor and activation of “silent synapses” [77, 78].

During the late phase of LTP, cGMP/PKG signaling causes release of calcium from ryanodine-sensitive stores, which phosphorylate CREB in parallel to cAMP/PKA signaling induced CREB phosphorylation, and in turn induces synaptic protein synthesis and synaptic structural changing [79-81].

### 4.3 cGMP and Neurogenesis

It has been demonstrated in an animal model of stroke that treatment of stroke with NO donor or the PDE5 inhibitor sildenafil increases brain cGMP levels and induces proliferation of progenitor cells in the subventricular zone (SVZ) and dentate gyrus as well as the number of immature neurons [82, 83]. An *in vitro *study using neurospheres isolated from SVZ indicates that the cGMP-enhanced neurogenesis occurs through PI3-K/Akt/GSK-3 (phosphoinositide 3-kinase/protein kinase B (aka AKT)/glycogen synthase kinase 3) pathway. Sildenafil caused a 4- to 6-fold increase in PKGII gene expression in neurospheres, which has implied a putative role of PKGII in cGMP-induced neurogenesis. Interestingly, clinical trials with PDE5 inhibitors indicate that these drugs might also have mood-enhancing effects as well as memory enhancing effects; although further studies are needed to determine whether these effects are related to increased cGMP levels and neurogenesis [84].

Cyclic GMP concentration relative to cAMP in neurons is involved in the modulation of dendritic and axonal guidance [85-87]. *In vitro* studies support the concept that the ratio of cAMP to cGMP sets the polarity of nerve growth-cone turning: high ratios favour attraction, whereas low ratios favour repulsion [86]. There is a reciprocal regulation between cAMP and cGMP in cultured hippocampal neurons; small alterations in one cyclic mononucleotide are physiologically relevant and yield reciprocal alterations in the other. Localised cAMP and cGMP activities in undifferentiated neurites promote and suppress axon formation, respectively, and exert opposite effects on dendrite formation [88]. Therefore the intricate interaction between cAMP and cGMP might contribute to integrate the new neurons into existent neurocircuits.

In our lab, we have found that 8-week treatment with fluoxetine and amitriptyline increased hippocampal cGMP content, with decreased hippocampal gene expression of PDE3, PDE4, and PDE5 and unchanged cAMP levels. We also found decreased hippocampal cAMP signaling, with unchanged cGMP levels and increased PDE3, PDE4, and PDE5 gene expression following chronic imipramine treatment. An understanding of the interconnectedness of cAMP and cGMP signaling pathways can be used to reconcile these seemingly divergent results. Our findings suggest that an increase of cGMP signaling pathway relative to cAMP signaling pathway in the hippocampus could be relevant to antidepressant action following chronic antidepressant administration. More research is needed to understand whether increases in hippocampal cGMP induced by chronic antidepressant treatment enhances neurogenesis and whether the compartmentalized interplay of cGMP and cAMP facilitates the incorporation of new neurons into the circuit.

### 4.4 NO and Serotonin Transporter (SERT)

NO has also been proposed to influence the activity of the serotonin reuptake transporter [89]. Selective serotonin reuptake inhibitor (SSRI) antidepressants are known to increase the amount of serotonin at the synapse by inhibiting serotonin transporter (SERT); therefore, SERT has been hypothesized as an important player in the pathophysiology of MDD. A recent proteomics based approach identified NOS1 as one of the proteins that physically interacts with the PSD-95/DISC large/ZO-1 (PDZ) domain of SERT [90]. HEK-293 cells transfected with NOS1 and YFP (yellow fluorescent protein)-tagged SERT were used to demonstrate that a physical interaction between these two proteins can be recapitulated in an *in vitro *cell culture model. Further, the interaction of NOS1 with SERT was found to decrease 5-HT uptake, indicating that SERT activity was decreased upon physical interaction with NOS1. In light of these results, NOS1 has been hypothesized as a putative endogenous antidepressant by decreasing the activity of SERT to increase extracellular 5-HT [59]. 

### 4.5 cGMP Signaling and MDD

#### 4.5.1 Genotyping SNPs in cGMP Signaling Components (NOS1 and PDEs)

Only one study has investigated the relationship between a specific NOS1 genetic variant (C2767) and MDD. The authors chose to study this genetic variant as a previous genetic study had found an association of this single nucleotide polymorphism (SNP) and schizophrenia [91]. In this study of 114 MDD patients treated with fluoxetine for 4 weeks, the NOS1 C2767 polymorphism was not significantly associated with susceptibility to MDD or to antidepressant treatment response when compared to 82 controls. However, the authors of that study have noted that 4 weeks fluoxetine treatment might not have been sufficiently long enough to achieve antidepressant treatment response and therefore they could not exclude the possibility that a longer duration of treatment could have yielded different results. Another genetic association analysis performed in a sample of Japanese patients with MDD and bipolar disorder failed to detect an association of 8 weeks fluvoxamine treatment with the rs41279104 or ex1c SNP in the NOS1 gene in MDD patients. The researchers acknowledged that using linkage disequilibrium and a larger sample size could yield different results [92].

Our lab has previously found that several SNPs in cGMP degrading PDE genes are related to the susceptibility to MDD and to antidepressant treatment response [93]. In that study, Mexican-American men and women MDD patients and controls were treated for 8 weeks with desipramine or fluoxetine and blood samples were taken for genotyping. Two SNPs in a cGMP specific PDE gene (rs729861 in PDE9A) and a dual substrate PDE gene (rs3770018 in PDE11A) were significantly associated with MDD at a Bonferroni corrected significance level of <0.0006 comparing control and depressed groups. Within the depressed group, two SNPs in dual substrate PDE genes (rs1549870 in PDE1A and rs1880916 in PDE11A) were associated with attained remitter and non-remitter status, indicative of antidepressant treatment response. Remission is defined as HAM-D (Hamilton Depression Rating Scale) score of less than 8. Although our findings indicating associations of SNPs in PDE1A, PDE9A, and PDE11A with MDD and antidepressant treatment response failed to reproduce in an independent sample of MDD patients versus controls (the sequenced treatment alternatives to relieve depression/STAR*D sample) [94, 95]. Recent post-mortem data support the disruption of PDE signaling system in the cerebellum of subjects with MDD, specifically, PRKG1 (protein kinase, cGMP dependent regulatory type 1) was upregulated [96]. The concept that genetic variations in cGMP-related PDEs could be relevant to susceptibility to MDD and to anti-depressant treatment response provides a novel target for anti-depressant drug development in the cGMP signaling system. 

#### 4.5.2 Behavioral Phenotype of cGMP Signaling Pathway knockout Mice

##### NOS1-/-, GCs-/-

NOS1 knockout mice display aggressive and impulsive behaviors [97, 98]. Other roles for NOS1 in the behavioral sensitization to cocaine [99], the behavioral response to inflammatory pain [100], and the development of social memory [101] have been proposed from behavioral assessments of NOS1 knockout mice. Knocking out either of the two α subunits of the GC enzyme, α1 or α2, in mice leads to absent LTP in the visual cortex, but the application of a cGMP analog combined with theta burst stimulation was capable restoring LTP [102].

##### PDE1B-/-, PDE10A-/-, PDE11A-/-

Mice deficient in the dual substrate PDE1B gene are reported to display hyperactivity when compared to wild-type mice in baseline measures of locomotor activity, with exaggerated hyperactivity in response to amphetamine and methamphetamine [103-105]. One study found additional defects in hippocampal spatial learning in PDE1B mutants, with an increase in path length to find a hidden platform in the Morris Water Maze [104]. Another study reported no significant differences in behavioral tests of anxiety-like behaviors (elevated plus maze), depressive-like behaviors (forced swim test), nociception (hot plate), or models of cognition (passive avoidance test and acquisition of conditioned avoidance response) in PDE1B knockout mice [103].

Behavioral phenotype assessment for mice deficient in the dual substrate PDE10A gene reveal decreased baseline locomotor activity, with decreased locomotor response to PCP and MK-801 as well as defects in the acquisition of the conditioned avoidance response [106, 107]. No significant differences were reported in behavioral tests of anxiety, depression, and nociception or in the passive avoidance and Morris Water Maze tasks of cognition [107].

Deletion of PDE11A results in a series of phenotypes associated with dysfunction of ventral hippocampus. The PDE11A-/- mice exhibit hyperactive locomotor behavior and increased sensitivity to NMDA receptor inhibitor MK 801. However, they tested normal in hippocampus-associated memory and anxiety/ depressive related behavior evaluations.

##### PKG/cGKII-/-

PKG/cGKII knockout animals showed enhanced anxiety-like behavior in the light-dark box test and elevated O-maze test. They were hyposensitive to the hypnotic effects of alcohol and consumed more alcohol [108].

#### 4.5.3 Behavioral Phenotypes with cGMP Signaling Pharmacotherapy

##### NOS Inhibitor 

Several studies have investigated the effects of NOS1 inhibition on behavioral tests of anxiety and depression. In these studies, NOS1 inhibition is achieved either through pharmacological means, using NOS1 inhibitors or *via* silencing the NOS1 gene. Results from these studies have led to the hypothesis that NOS1 inhibition has an antidepressant effect. Intra-hippocampal administration of the NOS1 inhibitor 7-nitroindazole decreases immobility time of rodents on the forced swim test [109]. Another NOS1 inhibitor, 1-(2-trifluoromethylphenyl)-imidazole (TRIM) can augment the effects of imipramine, citalopram, fluoxetine, and tianeptine on the forced swim test [110].

Male mice deficient in NOS1 were shown to display aggressive and impulsive behavior, thought to be related to decreased 5-HT receptor function and reuptake transporter activity [98]. Although chronic, 28 day treatment with the SSRI fluoxetine could not mitigate the anxiogenic phenotype of NOS1 knockout mice, hippocampal NOS1 expression was decreased in wild-type mice following chronic treatment with fluoxetine [97]. The chronic mild stress model of depression used in mice was shown to increase hippocampal NOS1 expression both acutely after 4 days and chronically after 21 or 56 days, with a coincident finding of decreased hippocampal neurogenesis in wild-type mice. In contrast NOS1 knockout mice were resistant to the effects of chronic mild stress on depressive-like behaviors and did not display decreased hippocampal neurogenesis [111]. In another study, chronic stress was once again shown to increase NOS1 and NOS2 expression in neocortex and hippocampus, with the administration of the non-specific NOS inhibitor N(omega)-nitro-L-arginine methyl ester (L-NAME) (10 mg/kg) increasing depressive-like behaviors [112].

##### PDE Inhibitors (PDEI)

Mice treated acutely with sildenafil displayed increased anxiety-like behaviors in the elevated plus-maze test, and this effect was reversed by pretreatment with the guanylyl cyclase (GC) inhibitor, methylene blue [92]. In contrast, another study in mice found that sildenafil decreased anxiety-like behaviors in the elevated plus-maze and open field tests of anxiety only when combined with high dose of L-arginine, and when sildenafil was given alone, it did not produce anxiety-like behaviors [113]. Sildenafil given chronically for seven days to rats in combination with atropine, a muscarinic receptor blocker displayed an antidepressant-like effect in the FST and combination treatment was as effective as chronic fluoxetine on decreasing immobility in the FST [8]. One study has reported an increase in aggressive behavior in mice one week following discontinuation of a chronic sildenafil treatment regimen (4 weeks, 10mg/kg sildenafil) [114]. Pretreatment with acute sildenafil has effects on behavioral tests of depression, preventing the decrease in immobility that normally occurs with the administration of acute adenosine [115], venlafaxine [11], and bupropion [116], but not of fluoxetine [117]. The results from studies on the effects of sildenafil in behavioral tests of depression and anxiety are complicated by differences in methodology including dosing regimens and behavioral tests. Different from the other cGMP signaling targeting drugs, the PDE2 inhibitors exhibit a univocal anxiolytic effect. It has been reported that PDE2 inhibitor Bay 60-7550 and ND7001 can induce anxiolytic effect in both stress and non-stressed mice, which can be antagonized by GCs inhibitor ODQ [118]. The oxidative-induced anxiety can also be reversed by Bay 60-7550 [119].

## 5 PHARMACOTHERAPY TARGETING cGMP SIGNALING (TABLE 1)

### 5.1 NO Donors

Although inhaled NO gas is used clinically to relieve pulmonary hypertension, NO gas is unfortunately highly reactive and can quickly oxidize to nitrogen dioxide. NO donor drugs resolve this issue by acting as carriers for NO, providing stability until appropriate release of the NO molecule is desired. There are three major classes of NO donor drugs: organic nitrates, diazeniumdiolates and S-nitrosothiols. The organic nitrates are the only NO donor drugs that are currently approved for clinical use and include the heart medications nitroglycerin and sodium nitroprusside (SN). Nitroglycerin, also known as glyceryl trinitrate (GTN), is used to acutely relieve angina pectoris (chest pain) and SN is used in the setting of a hypertensive crisis to quickly decrease blood pressure. The diazeniumdiolates, also known as NONOates, are composed of a diolate group bound *via* a nitrogen atom to a nucleophile adduct. The diazeniumdiolates are at present used experimentally in models of cardiovascular disease and include diethylamine NONOate (DEA/NO), spermine NONOate (SPER/NO), PROLI/NO, V-PYRRO/NO, and JS-K/NO. S-nitrothiols, also known as thionitrites, are composed of a thiol connected to a NO moiety by a single chemical bond. The S-nitrothiols are also currently used experimentally in models of cardiovascular disease include S-nitroso-glutathione (GSNO), S-nitroso-N-acetylpenicillamine (SNAP) and S-nitroso-N-valeryl-penicillamine (SNVP) [120].

### 5.2 sGC/NO Receptor Agonists

Several stimulators or activators of the soluble GC enzyme (sGC), developed by Bayer Healthcare (Leverkusen, Germany), are currently being tested in experimental and clinical settings. The sGC stimulator drugs include BAY 41-2272, BAY 41-8543 and BAY 63-2521 (riociguat). Riociguat is currently being tested in clinical trials for the treatment of chronic thromboembolic pulmonary hypertension as well as in pulmonary arterial hypertension [121]. The sGC activator drug BAY 58-2667 (cinaciguat) is currently being tested in clinical trials for its use in the treatment of acute decompensated heart failure [122]. 

### 5.3 PDE Inhibitors (PDEI)

There is growing interest in the clinical utility of PDE inhibitor (PDEI) drugs as these pharmacologic agents can increase levels of cyclic nucleotides to influence cell responses [123, 124]. Some PDEI drugs demonstrating selective inhibition of PDE3 or PDE5 enzymes are approved for clinical use, and while inhibitors for other PDEs can be acquired, most of these agents are still in development. PDE1Is include MMPX and vinpocetine. PDE2Is include BAY 60-7550 and EHNA. PDE3Is include amrinone/inamrinone, anagrelide, cilostamide, cilostazol, enoximone, milrinone, siguazodan, and trequinsin. Anagrelide (Agrylin®) and cilostazol (Pletal®) are used clinically to treat thrombocytopenia and peripheral vascular disease, respectively [125, 126]. Amrinone/inamrinone (Inocor®) and milrinone (Primacor®) are used to treat congestive heart failure [127]. Treatment with PDEIs is hypothesized to have a neuroprotective effect; in one *in vitro *study, 5-10 μM trequinsin (PDE3I) and vinpocetine (PDE1I) was able to prevent the neurotoxic effects of different models of cell injury following their application in rat cortical cell culture. It was hypothesized that the neuroprotective of these PDEIs could occur through the suppression of the cell cycle element cyclin D1 and the pro-apoptotic factor caspase 3 [128].

PDE5Is include acetildenafil, avanafil, icariin, lodenafil, mirodenafil, MY-5445, sildenafil, T-0156, tadalafil, thiomethisosildenafil, udenafil, and vardenafil. Sildenafil (Viagra®), tadalafil (Cialis®) and vardenafil (Levitra®) are used clinically to treat erectile dysfunction; sildenafil (Revatio®) is also used to treat pulmonary hypertension [119]. PDE9Is include BAY 73-6691 and SCH-51866 [62]. PDE10Is include papaverine and PF-2545920. There are currently no inhibitors demonstrating selectivity for PDE6 or PDE11. 

The PDE5 inhibitor sildenafil competitively inhibits the binding pocket of the catalytic domain of PDE5 so that cGMP is unable to bind and become hydrolyzed to 5’GMP; blockade of PDE5 by sildenafil therefore results in increased cGMP levels [124]. Sildenafil is administered orally in tablet formulations of the following doses: 25 mg, 50 mg or 100 mg. The bioavailability of sildenafil following oral administration is 41% with metabolism or elimination primarily by the hepatic enzymes, CYP3A4 and CYP2C9 [129]. The absorption of sildenafil reveals that peak plasma concentration is reached within 60 minutes after administration, with therapeutic drug levels from 200ng/mL to 440 ng/mL, corresponding to 0.4 µM and 1 µM, respectively. In the steady state, sildenafil displays a mean volume of distribution of 105 L, indicative of distribution into tissues. 

## 6 CONCLUDING REMARKS

**Fig. (1):**
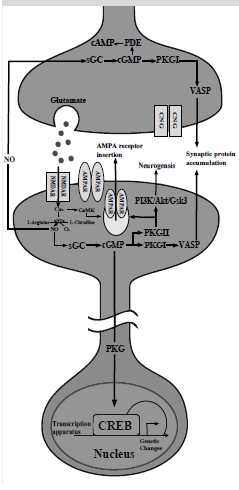
The cGMP signaling pathway. Presynaptic (top) and postsynaptic (bottom) elements of the cGMP signaling cascade are represented. AMPAR = 2-amino-3-(5-methyl-3-oxo-1,2-oxal-4-yl)propanoic acid receptor; CaMK = calcium-calmodium-dependent kinase; cAMP = cyclic adenosine monophosphate; cGMP = cyclic guanosine monophosphate; CNG = cyclic nucleotide gated channels; NO = nitric oxide; nNO = neuronal NO; PDE = phosphodiesterase; PI3K/AKT/Gsk3 = phosphoinositide 3-kinase/protein kinase B (aka AKT)/glycogen synthase kinase 3; PKGI = cGMP dependent protein kinase I; PKGII = cGMP dependent protein kinase II; sGC = soluble guanylate cyclase; VASP = vasodilator-stimulated phosphoprotein.

The search for mechanisms of antidepressant action has led to the investigation of changes in neuroplasticity following antidepressant treatment. While the cAMP signaling cascade in the cortex and hippocampus has received the most attention todate, accumulating evidence in rodents and humans suggests a role for cGMP signaling in stress induced disturbance of neuroplasticity and antidepressant action as well. In this review we have summarized the biology of cGMP signaling cascade in the brain and emergent data supporting the role of NO/cGMP signaling in neuroplasticity and MDD. We suggest that functional synaptic plasticity starts with activation of glutamate system in the CNS; furthermore, a growing body of evidence implicates a role of the glutamatergic system in the pathophysiology and treatment of MDD [130]. Activation of glutamate receptors increases in Ca^2+^ levels at the postsynaptic neuron and stimulates NO synthesis, which stimulates the NO/cGMP pathway. NO can also act transynaptically at the presynaptic neuron or surrounding cells. The presynaptic and postsynaptic cGMP signaling enhances synaptic transmission (Fig. **1**), induces synaptic protein synthesis during the late phase of LTP and might facilitate neurogenesis through activation of several downstream substrates. It is beyond the scope of this review to discuss the immune/inflammatory aspects of NO and cGMP signaling pathway through the role of inducible nitric oxide synthase (NOS2).

## Figures and Tables

**Table 1:** Pharmacological Therapy Targeting the cGMP System

Type	Drugs		Effects

NO donors	Organic nitrate	Nitroglycerin (GTN)	Cardiovascular disease
		Sodium nitroprusside(SN)	

	diazeniumdiolate	Diethylamine NONOate (DEA/NO)	Cardiovascular disease
		Spermine NONOate (SPER/NO)	
		PROLI/NO, VPYRRO/NO, JS-K/NO	

	S-nitrosthiols	S-nitrosoglutathione(GSNO)	Cardiovascular disease
		S-nitroso-N-acetylpenicillamine(SNAP)	
		S-nitroso-N-valerylpenicillamine(SNVP)	

sGC/NO receptor agonist		BAY 41-2272, BAY 41-8543,BAY 63-2521, BAY 2667	Cardiovascular disease

PDE inhibitors	PDE1I	MMPX, vinpocetine	Prevent neurotoxic effects

	PDE2I	BAY 60-7550, EHNA	Anxiolytic effect

	PDE3I	Amrinone, milrinone	Congestive heart failure

		anagrelide, cilostamide, cilostazol, enoximonesiguazodan,	Thrombocytopenia and peripheral vascular disease

		trequinsin	Prevent neurotoxic effects

	PDE5I	Sildenafil, tadalafil, vardenafil	Erectile dysfunction and pulmonary hypertension

	PDE9I	BAY 73-6691, SCH-51866, PF-4181366	Improve learning and memory

	PDE10I	Papaverine, PF-2545920	
